# A systematic review of screening instruments for depression for use in antenatal services in low resource settings

**DOI:** 10.1186/s12888-017-1273-7

**Published:** 2017-03-24

**Authors:** Genesis Chorwe-Sungani, Jennifer Chipps

**Affiliations:** 1 School of Nursing, University of the Western Cape, Private Bag X17, Bellville, 7535 South Africa; 2 Kamuzu College of Nursing, University of Malawi, Blantyre, Malawi; 3 Honorary Affiliate, Sydney Nursing School, University of Sydney, Sydney, Australia

**Keywords:** Depression, screening instrument, antenatal, EPDS, Low resource setting

## Abstract

**Background:**

In low resource settings, short, valid and reliable instruments with good high sensitivity and specificity are essential for the screening of depression in antenatal care. A review of published evidence on screening instruments for depression for use in antenatal services in low resource settings was conducted. The aim of this review was to appraise the best available evidence on screening instruments suitable for detecting depression in antenatal care in low resource settings.

**Methods:**

Searching, selection, quality assessment, and data abstraction was done by two reviewers. ScienceDirect, CINAHL, MEDLINE, PubMed, SABINET and PsychARTICLES databases were searched using relevant search terms. Retrieved studies were evaluated for relevancy (whether psychometric data were reported) and quality. Data were synthesised and sensitivity and specificity of instruments were pooled using forest plots.

**Results:**

Eleven articles were included in the review. The methodological quality ranged from adequate to excellent. The review found 7 tools with varying levels of accuracy, sensitivity and specificity, including the Edinburgh Postnatal Depression Scale, Beck Depression Index, Centre for Epidemiologic Studies Depression Scale 20, Hamilton Rating Scale for Depression, Hopkins Symptoms Checklist-25, Kessler Psychological Distress Scale and Self-Reporting Questionnaire. The Edinburgh Postnatal Depression Scale was most common and had the highest level of accuracy (AUC = .965) and sensitivity.

**Conclusion:**

This review suggests that the Edinburgh Postnatal Depression Scale can be a suitable instrument of preference for screening antenatal depression in low resource settings because of the reported level of accuracy, sensitivity and specificity.

**Prospero registration:**

CRD42015020316.

## Background

Depression is a major health problem affecting pregnant women in low resource settings [1, 2] with high prevalence rates of antenatal depression (10.7 to 47%) [1–4]. Antenatal depression can lead to poor uptake of antenatal care, adverse birth outcomes [3] and is a risk factor for postnatal depression [5]. Routine screening for antenatal depression is essential for early identification of pregnant women with depressive symptoms [6] and routine antenatal contacts with health providers provide opportune times for assessing, preventing and treating depression during pregnancy [7].

There are however some challenges in these settings as many women may be ashamed to speak about depression as there is a cultural expectation of pregnancy happiness. In addition, these settings are understaffed, lack consultation rooms, have heavy workloads with high midwife to pregnant woman ratios. Midwives commonly have limited consultation time to explore depressive symptoms or risk factors and often lack guidelines or tools for assessing psychosocial status of pregnant women [8]. In this setting, screening instruments suitable for the early detection of depression must be effective in the identification of individuals who are cases and those who are not [9]. Suitable instruments must therefore demonstrate both high sensitivity and specificity [9].

Many validation studies for depression screening tools have previously been conducted in high income countries (HICs) whose cultures and socio-economic context differ from those in low resource settings. Due to a concern about the variation of performance of screening tools in different populations and settings [10] and with the aim of identifying a tool suitable to be recommended for use in antenatal services in low resource settings, a systematic review of instruments for screening depression in antenatal care in low resource settings was conducted.

## Methods

The Standards for the Reporting of Diagnostic Accuracy Studies (STARD) guidelines were used to conduct the review [10].

### Search process

A limited search of the Cumulative Index of Nursing and Allied Health Literature (CINAHL) and Medline was undertaken to identify relevant keywords contained in the title, abstract, and subject descriptors. Search terms and synonyms were then identified for use in searching different databases for screening studies conducted in antenatal clinics in low resource settings. Low resource settings refer to settings where health care systems do not meet the minimum standards set by the World Health Organisation (WHO) or any other quasi-governmental organisation [11]. In this review, low resource settings were defined as health care settings synonymous with those found in low income and lower middle income countries as defined by World Bank [12] and some health care settings in upper middle income countries (UMICs), such as South Africa, where disparities in the public health infrastructure or supplies or human resources [13] are found. Some articles from low resource settings are not indexed to indicate that they are reporting about health outcomes or disparities for under-served populations in low resource settings [14] and the term, ‘low resource settings’, was not included in the search terms but applied manually at the article review stage. Date limits were set from 2000 to 2015 in anticipation that a wider period to be searched will yield many relevant studies with recent evidence. Detailed search terms are supplied in Table 1.

**Table 1:** Search terms

Data base	Terms used
ScienceDirect	ALL (“screening instruments” OR “screening tools” OR “screening scale”) and ALL (depression AND antenatal).
	ALL (“screening instruments” OR “screening tools” OR “screening scale”) and ALL (depression AND pregnancy OR prenatal) AND LIMIT-TO (topics, “woman, patient, depression, depression scale, pregnancy, mental health, depressive symptom, health care, maternal, adolescent, health”).
	ALL (EPDS or CESD-10 or HSCL or K-6 or K-10 or SRQ or PHQ or GHQ) and ALL (depression AND antenatal) AND LIMIT-TO(topics, “woman, pregnancy, obstet gynecol, depression scale, depression, health, patient, maternal, depressive symptom, mental health”).
	ALL (“screening instruments” OR “screening tools” OR “screening scale”) and ALL (depression or “depressive disorder” AND antenatal or prenatal)
CINAHL	TI screening AND TI depression AND TI pregnancy
	screening AND depression AND pregnancy AND LIMIT-TO (research article)
	screening tools AND depression AND antenatal
	epds validity AND depression AND antenatal
	TI Edinburgh postnatal depression scale OR TI Hopkins symptom checklist OR TI self-report questionnaire OR TI center for epidemiological studies depression scale OR TI patient health questionnaire OR TI general health questionnaire OR TI beck depression inventory OR TI whooley questions AND TI antenatal AND LIMIT-TO (research article)
MEDLINE	TX depression AND TX screening tools AND pregnant women
	TI screening test AND TI antenatal depression
	TX depression AND TX screening AND TX pregnant women
	TI prenatal depression AND TI screening
Pubmed	((((“screening instruments”) OR “screening tools”) OR “screening scales”) AND depression) AND antenatal
	((screening[Title]) AND depression[Title]) AND antenatal[Title]
	(((screening[Title]) AND depression[Title]) AND pregnancy[Title])
SABINET	(alltext:(depression AND screening)^20 AND alltext:(antenatal)^20)
	(alltext:(depressive AND disorder AND screening)^20 AND alltext:(pregnant AND women)^20)
PsychARTICLES	depression AND screening AND pregnancy

The following databases were searched: ScienceDirect, CINAHL, MEDLINE, PubMed, SABINET and PsychARTICLES and results were imported into Endnote. Reference lists of key articles identified were hand searched to identify further relevant articles. Manual searches of indexes and “grey” literature databases were not carried out. The preliminary searches were conducted between August and September 2015 and the final search was done on 4th September 2015.

### Review process, selection and data extraction

After the initial search, duplicates and irrelevant articles (conferences, congresses, editorials, commentaries, reviews, news, old) in the Endnote database were removed and the search data were exported to Excel. Articles for review were then selected in three phases.

#### Abstract and title screening

In this phase, the reviewers scanned the identified titles and abstracts independently and indicated in the Excel database which articles were relevant. Where the abstract did not provide enough information or the reviewers were unsure, the full text articles were reviewed and agreement reached between the reviewers on the inclusion or exclusion of the article. A kappa statistic was calculated to assess the level of agreement for eligibility for inclusion at this stage.

#### Screening based on PICOS criteria

The second phase of selection consisted of a review of articles by applying and extracting the PICOS criteria: Participants (P) (pregnant women at any stage of pregnancy attending antenatal care), Index test (I) (Screening instrument), Comparator test (C) (gold standard- psychiatric assessment), Outcome measures (O) (psychometric properties of screening instrument) and study setting (S) (low resource settings). In this phase, articles from HICs were excluded. Full text articles from UMICs were reviewed and included if the study setting was a public health setting and the studies were located in low resource settings where disparities in the public health infrastructure or supplies or human resources in the services were adequately described.

#### Article review

In the third phase, full texts of the articles were reviewed for reported validity of one or a combination of depression screening instruments (sensitivity, specificity, area under curve [AUC]) and whether a gold standard was present. The articles were independently examined by the reviewers to confirm inclusion. The gold standard was set as a formal diagnostic psychiatric assessment of depression as the most accurate test to detect the presence or absence of depression [15]. Psychiatric diagnostic assessment of depression included the use of the Structured Clinical Interview for DSM-IV (SCID), the Mini-International Neuropsychiatric Interview (MINI), Composite International Diagnostic Interview (CIDI), International Classification of Diseases version 10 (ICD-10) or the Diagnostic and Statistical Manual of Mental Disorders version 4 (DSM-IV) by a psychiatrist to assign a diagnosis. The MINI and SCID are compatible with DSM-IV and have sensitivity/specificity above minimum acceptable level (.8/.8) for structured interviews which are used as gold standards [16]. Instruments that are routinely used for depression screening such as Edinburgh Postnatal Depression Scale (EPDS) or other nonconventional psychiatric assessment instruments were not considered as gold standards.

Eligibility for full article review, assessment of study characteristics, and relevant data extraction was conducted using a review tool in Excel that included the PICOS criteria and the confirmation of the presence of psychometrics and a gold standard. For each eligible study the reviewers extracted information concerning: author, country of study, sample, gold standard, screening instrument, Area under the Curve (AUC), sensitivity (Se) and specificity (Sp). All results were subject to double data entry.

### Assessment of methodological rigour

The Quality Assessment of Diagnostic Accuracy Studies (QUADAS) [17] was used by both reviewers to assess the psychometric quality of the final selected articles. The QUADAS has 14 items with three possible responses ‘Yes’, ‘No’ and ‘Unclear’. In the QUADAS, the target condition was depression during pregnancy, the index test was a screening instrument used to screen for depression, and the reference standard was the gold standard against which the index test was validated. The QUADAS items measure the variability of study samples (items 1–2), methodological rigor and bias (items 3–7, 10–12 and 14), and the quality of reporting methodology (items 8, 9 and 13). The scoring of QUADAS is not standardised [18] but studies were categorised as ‘excellent’ (11 to 14 items), ‘good’ (9 to 10 items), ‘adequate’ (6 to 8 items), ‘poor’ (4 to 5 items) or ‘unacceptable’ (0 to 3 items) based on the number of items that were answered ‘Yes’ [17].

### Analysis

Descriptive data extraction and presentation was done to compare screening instruments’ psychometrics data in a between-study literature analysis [19]. A meta-analysis was conducted using REVMAN by pooling individual and all instruments sensitivity and specificity data to show the pooled ability of the screening instruments to identify depression. Upper and lower confidence intervals (95%) for sensitivity and specificity of screening instruments were calculated.

## Results

### Search and review results

**Fig. 1:**
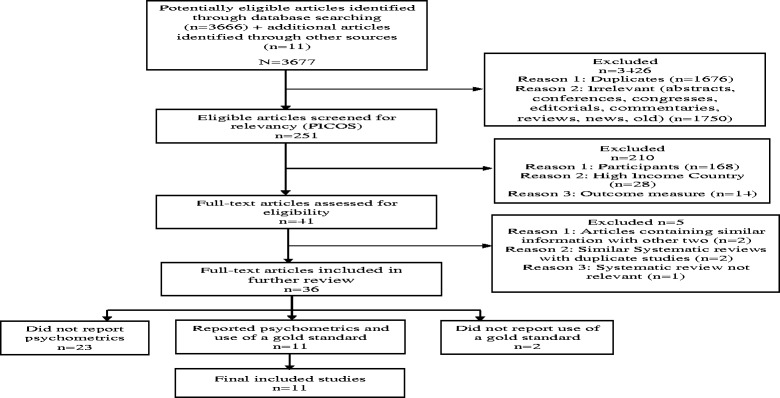
Study flow diagram based on STARD

The electronic search yielded 3666 published articles (Fig. 1). Eleven (11) additional articles were sourced from authors on ResearchGate and reference lists of full text articles resulting in a total number of 3677 published articles. A total of 1676 duplicates were removed leaving 2001 articles. Irrelevant articles consisting of conferences, congresses, editorials, commentaries, reviews, news and old articles (≤ 1999) were removed (*n* = 1750), leaving 251 articles. The 251 articles which remained were then screened for relevancy by the reviewers using the PICOS criteria, excluding a further 210 articles [Participants (*n* = 133), Outcome (*n* = 21) and HICs articles (*n* = 28)], leaving 41 articles (38 primary research studies and 3 systematic reviews). The reviewers’ ratings were in agreement with a Kappa = .97.

The systematic reviews (*n* = 3) were excluded after being screened for relevancy for inclusion in this review. One systematic review [20] focused on the efficacy of antenatal group interventions aimed at reducing postnatal depression in at risk women. This systematic review did not report any validity data of the depression screening instruments and thus was excluded. The second systematic review by Akena and colleagues [21] examined the accuracy of depression screening instruments validated in general health settings in low and middle income countries (LMICs). This systematic review included three studies conducted in antenatal settings [4, 22, 23] which also had been identified as part of the 38 articles for primary studies in our review. The third systematic review focused on the reliability and validity of instruments for screening perinatal depression in African settings [24]. This systematic review included eight articles for studies which were conducted in antenatal settings of which four [3, 25, 26] were included in the 38 primary articles in our review. The other four articles [27–30] were published before 2000 and were excluded due to the time limits of the search terms. Further review of the full texts of the 38 articles showed that two pairs of articles [25, 31] and [3, 26]] reported the same data from two different studies and one article from each pair was retained resulting in 36 articles included for further review.

### Selected studies for full text review (*n* = 36)

The study characteristics of the 36 selected studies for further review are provided in Table 2. The majority of the studies were published between 2010 and 2015 and only one study was published in a nursing journal. Most of the articles (*n* = 18) were cross sectional prevalence studies and five (*n* = 5) were psychometric validation studies measuring reliability and validity of screening instruments. In reviewing these studies for reported psychometrics of sensitivity, specificity, Area under the curve and the relevant gold standards, two studies [32, 33] were excluded (no gold standard as defined by this study) and a further 23 studies were excluded due to inadequate reporting of psychometrics. One third of the articles (*n* = 11) reported psychometrics and a gold standard and met the final selection criteria for inclusion in the review (Table 2).

**Table 2:** Characteristics of 36 studies considered for review

Characteristics	*n* = 36(100%)	*n* = 11(100%)
Year of publication		
2000–2009	12(33.3)	3(27.3)
2010–2015	24(66.7)	8(72.7)
Upper Middle Income Country		
Brazil	7(19.4)	2(18.2)
China	1(2.8)	0(0)
Iran	1(2.8)	0(0)
Jamaica	1(2.8)	0(0)
Peru	2(5.6)	0(0)
South Africa	6(16.7)	2(18.2)
Thailand	1(2.8)	0(0)
Turkey	2(5.6)	0(0)
Mexico	3(8.3)	2(18.2)
Lower Middle Income Country		
India	1(2.8)	1(9.1)
Pakistan	2(5.6)	1(9.1)
Sri Lanka	1(2.8)	0(0)
Low Income Country		
Malawi	2(5.6)	1(9.1)
Tanzania	4(11.1)	1(9.1)
Nepal	1(2.8)	0(0)
Uganda	1(2.8)	1(9.1)
Study type		
Validation	5(13.9)	5(45.5)
Epidemiological	4(11.1)	0(0)
Cross sectional	18(50)	4(36.3)
Randomized controlled trial	3(8.3)	1(9.1)
Descriptive	1(2.8)	0(0)
Prospective	3(8.3)	1(9.1)
Ethnography	1(2.8)	0(0)
Naturalistic	1(2.8)	0(0)
Journal type		
Medicine	33(91.6)	11(100)
Nursing	1(2.8)	0(0)
Multidisciplinary	1(2.8)	0(0)
Social and behavioural sciences	1(2.8)	0(0)
Se, Sp, AUC, Gold standard reported	11(30.6)	11(100)

*AUC* area under curve, *Se* sensitivity, *Sp* specificity

### Findings from studies for inclusion in review (*n* = 11)

All 11 articles were published in medical journals, mostly from 2010 onwards (*n* = 8). A number of articles were validation studies (*n* = 5) that reported psychometrics (reliability and validity). There were also 4 cross sectional prevalence studies (*n* = 4), one prospective study and one randomised trial. These last-mentioned 6 studies generally reported on prevalence of prenatal depression and risk factors but included psychometric properties of the screening instruments. All the screening instruments reported in the selected articles were adapted by translating them to local languages in each setting.

### Quality of reviewed studies

All 11 articles were rated for quality by both reviewers. Overall the quality was satisfactory with six articles [1, 23, 25, 34–36] rated as excellent, three [37–39] good and two [3, 4] adequate. All the articles clearly described the selection criteria for the sample and reported the index test as independent of the gold standard. All articles, except one [39], regardless of overall quality, used random samples. The two articles rated as ‘adequate’ [3, 4] did not sufficiently report the execution of a gold standard and it was difficult to ascertain whether individuals who administered index tests or gold standards were blinded to each other’s results. Articles with ‘excellent’ quality were the psychometric validation studies and the randomised controlled trial.

### Screening instruments used in antenatal care in low resource settings

The articles included seven (*n* = 7) screening tools, namely the Beck Depression Index (BDI), Centre for Epidemiologic Studies Depression Scale (CES-D)-20, Edinburgh Postnatal Depression Scale (EPDS), Hamilton Rating Scale for Depression (HAM-D), Hopkins Symptoms Checklist (HSCL)-25, Kessler Psychological Distress Scale (K-10) and Self-Reporting Questionnaire (SRQ) that were used for screening antenatal depression in low resource settings (Table 3). The BDI and HAM-D are not normally used for diagnostic purposes or screening purposes but to estimate the severity of depression for the past 3 or 7 days. EPDS was designed for use in postnatal period and it has been investigated for antenatal use as well.

**Table 3:** Results of included studies (*n* = 11)

Author	Country of study	Type of study	Sample (n)	Gold standard	Screening Instrument	AUC (95% CI)	Se	Sp
Adewuya et al. (2006) [25]	Nigeria	Validation study	182 pregnant women (32–36 weeks)	MINI	EPDS	.965	.867	.915
Alvarado-Esquivel et al. (2014a) [36]	Mexico	Validation study	158 adult pregnant women (2-9 months)	DSM-IV	EPDS	.810	.757	.744
Alvarado-Esquivel et al. (2014b) [37]	Mexico	Validation study	120 teenage pregnant women (3–9 months)	DSM-IV	EPDS	.890	.704	.849
e Couto et al. (2015) [1]	Brazil	Validation study	247 pregnant women (2nd trimester)	MINI	EPDS	.850	.816	.733
					BDI	.900	.820	.846
					HAM-D	.860	.877	.746
Fernandes et al. (2011) [4]	India	Cross sectional study	194 pregnant women (3rd trimester)	MINI	EPDS	.950	1.00	.849
					K-10	.950	1.00	.813
Kaaya et al. (2002) [23]	Tanzania	Randomized controlled trial	903 HIV positive pregnant women (8–26 weeks)	SCID	HSCL-25	.860	.890	.800
Martins et al. (2015) [39]	Brazil	Cross sectional study	807 adolescent pregnant women (2nd trimester)	MINI	EPDS	.890	.811	.827
					BDI	.870	.867	.738
Natamba et al. (2014) [35]	Uganda	Cross sectional study	123 [36 HIV positive and 87 HIV negative pregnant women] (10–26 weeks)	MINI	CES-D-20	.820	.727	.785
Rochat et al. (2013) [3]	South Africa	Cross sectional study	109 [49 HIV positive and 60 HIV negative pregnant women] (Second half of pregnancy)	SCID	EPDS	.817	.690	.780
Spies et al. (2009) [22]	South Africa	Prospective study	129 pregnant women (<20 weeks)	SCID	K-10	.660	.730	.540
Stewart et al. (2013) [34]	Malawi	Validation study	224 pregnant women (28–34 weeks)	SCID	EPDS	.811	.688	.795
					SRQ	.833	.763	.813

*AUC* area under curve, *BDI* beck depression index, *CES-D* centre for epidemiologic studies depression scale, *CI* confidence interval, *DSM-IV* diagnostic and statistical manual of mental disorders version 4, *EPDS* Edinburgh postnatal depression scale, *HAM-D* Hamilton rating scale for depression, *HSCL-25* Hopkins symptoms checklist 25, *K-10* Kessler psychological distress scale 10, *MINI* mini-international neuropsychiatric interview, *SCID* structured clinical interviews for DSM IV axis 1 diagnoses, *SRQ* self-reporting questionnaire, *Se* sensitivity, *Sp* specificity, [ ] number in reference list, *HIV* human immunodeficiency virus

**Fig. 2:**
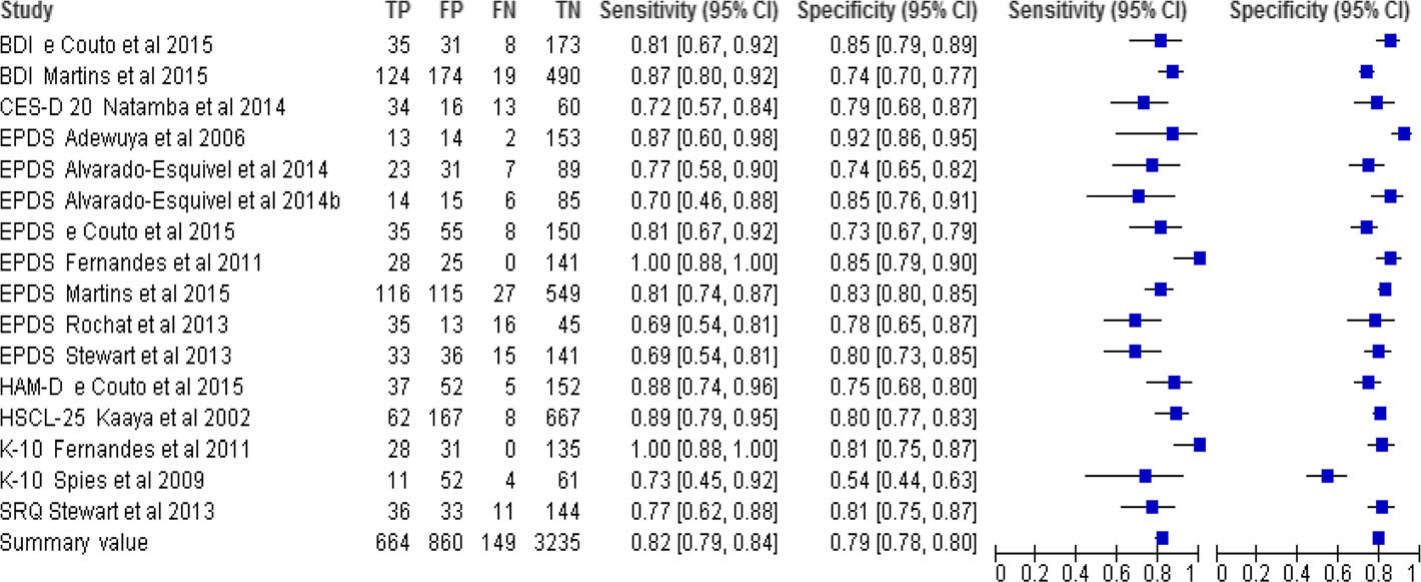
Sensitivity and specificity of selected tools. BDI=Beck Depression Index, CES-D=Centre for Epidemiologic Studies Depression Scale, EPDS= Edinburgh Postnatal Depression Scale, HAM-D=Hamilton Rating Scale for Depression, HSCL 25=Hopkins Symptoms Checklist 25, K10=Kessler Psychological Distress Scale 10, SRQ 20=Self-Reporting Questionnaire 20, FN=False negative, FP=False positive, TN=True negative, TP=True positive

**Fig. 3:**
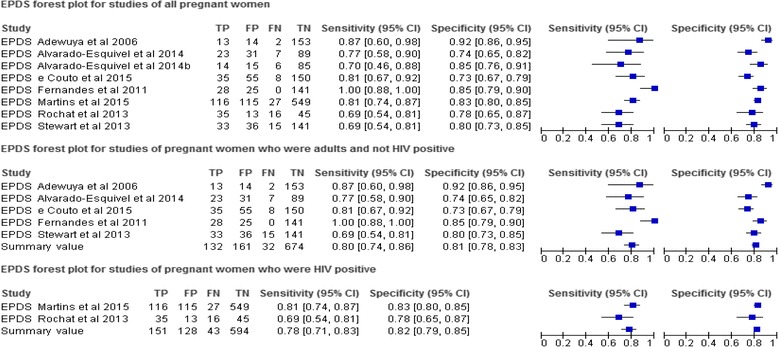
Forest plot of EPDS studies. EPDS= Edinburgh Postnatal Depression Scale, FN=False negative, FP=False positive, TN=True negative, TP=True positive

**Fig. 4:**
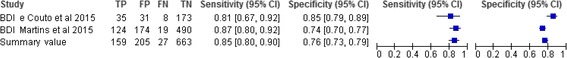
Forest plot of BDI studies. BDI=Beck Depression Index, FN=False negative, FP=False positive, TN=True negative, TP=True positive

**Fig. 5:**
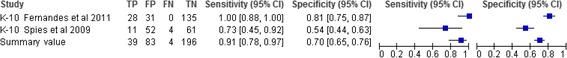
Forest plot of K-10 studies. K10=Kessler Psychological Distress Scale 10, FN=False negative, FP=False positive, TN=True negative, TP=True positive

Seven studies (*n* = 7) used a single screening instrument while four (*n* = 4) used a combination of two or three instruments. The EPDS was the most widely used instrument (8 studies), followed by the BDI and K-10 (2 studies each). The MINI was the most widely used gold standard being used in five of the 11 studies. In assessing the accuracy of screening instruments in detecting depression among pregnant women, an AUC score range is classified as low (.500 to .700), moderate (>.700 to .900) and high (>.900) [40]. The EPDS had the highest level of accuracy (AUC = 0.965) while K-10 had the lowest level of accuracy (AUC = .660). The BDI, CES-D, HAM-D, HSCL-25 and SRQ had moderate accuracy with AUC ranges from .820 to .900. A forest plot showed that the included studies were heterogeneous because error bars for sensitivity and specificity plots did not include the summary values-sensitivity of .82 and specificity of .79 (Fig. 2). As such 5 distinct subgroups based on participants or type of instrument were formulated and graphical test using forest plots showed that one EPDS studies subgroup of all pregnant women was heterogeneous while other four were homogeneous (Figs. 3, 4 and 5). Schriger and colleagues recommended that a forest plot should consist of a minimum of two studies and discourages conducting heterogeneity tests when there are less than five studies [41].

#### The EPDS

The EPDS is a 10-item self-reported questionnaire about feelings of depression experienced in the postnatal period rated over the past 7 days with each item being rated on four exclusive scores that range from 0 to 3 [42]. The EPDS is shorter compared to other instruments (BDI, CES-D-20, HSCL-15 and SRQ) and takes about 5 min to complete.

The sensitivity and specificity of EPDS differed across studies which may be attributed to variations in study methodologies [43] and characteristics of populations under study [1]. The sensitivity of the EPDS across the 8 studies ranged from Se = .688 to Se = 1, with a specificity from Sp = .733 to Sp = .915. EPDS had pooled sensitivity of. 80 and pooled specificity of .81 after excluding studies for pregnant women with Human Immunodeficiency Virus (HIV) [3] and those who were young [37, 39] (Fig. 3). Pooling was done in these two EPDS studies subgroups because they were considered to be sufficiently homogeneous in terms of participants, screening instrument and outcomes [44]. The EPDS had the highest level with an AUC ranging from .770 to .965 indicating a high level of accuracy in detecting depression in pregnant women in low resource settings.

#### The BDI

The BDI is a 21-item self-rating inventory which measures symptoms of depression on a scale from 0 to 3 [45]. Sensitivity of BDI in the two studies was Se = .867 and Se = .82 with AUC of .87 and .90 respectively (Table 3) BDI had pooled Se = .85 and pooled Sp = .76 (Fig. 4).

#### K-10

The Kessler-10 (K-10) is a self-administered 10-item questionnaire which measures anxiety and depression rated over the past 4 weeks [46]. The data from the two K-10 studies were inconsistent with the second highest accuracy (AUC = .95) in India and the lowest accuracy (AUC = .66) in South Africa and the highest sensitivity (Se = 1.0) in India and lowest specificity (Sp = .54) in South Africa (pooled Se = .91 and pooled Sp = .70) (Fig. 5).

#### Other instruments

A number of other screening instruments were also reported as having been used in low resource settings. These were: CES-D, a 20 item self-rating scale which measures depressive symptomatology in the general population [47]; the HSCL-25, a self-report inventory for identifying common psychiatric symptoms [48] which include fifteen items for screening depression (HSCL-15); the SRQ, a 20 item scale that is used to assess for psychiatric disturbance [49] and the HAM-D, a 21 items clinician administered scale that assesses severity of, and change in, depressive symptoms [50].

## Discussion

An instrument being considered for selection for routine screening, should be inexpensive, be easy to administer, cause minimal discomfort and have high reliability and validity in distinguishing between cases and non-cases of a condition [51]. In this review, screening instruments with a pooled sensitivity/specificity balance >85% were considered as ideal to distinguish between depressed and non-depressed women. The EPDS met criteria for both brevity and validity with this review, similar to two earlier systematic reviews [21, 24] which found high sensitivity, high specificity and the highest level of accuracy (AUC = .965). Though the K-10 had the best pooled sensitivity (Se = .91), the EPDS had the best pooled specificity (Sp = .81). The BDI had a good sensitivity/specificity balance (Se = .85 and Sp = .76) respectively, but the EPDS sensitivity/specificity balance was more ideal with a higher specificity (important in screening out non-cases) and adequate sensitivity (Se = .80).

A second finding from this review is evidence that seven local language versions of depression screening instruments (BDI, CES-D-20, EPDS, HAM-D, HSCL-25, K-10 and SRQ) had acceptable sensitivities or specificities and level of accuracy in antenatal clinics in low resource settings. However, none of these instruments were specifically designed to measure antenatal depression in low resource settings and their sensitivity and specificity varied with studies. The included studies had significant differences in methodology, population sampled, gestation period, type of instrument used and gold standards which indicated that there was clinical heterogeneity amongst included studies. Nevertheless, forest plots showed that distinct subgroups of studies which used similar participants and instruments were homogeneous. But one has to bear in mind that this method of identifying heterogeneity has limited power in detecting bias when studies are few [52].

It is documented that HIV prevalence in a population may influence the prevalence and severity of depression [3]. However, in this review, the instruments (EPDS and K-10) which had highest sensitivity (Se = 1.0) were validated in general population of pregnant women while lowest sensitivity (Se = .69) of EPDS was found in both general population of pregnant women, and in sample comprising of HIV positive and HIV negative pregnant women. In this review, it was clear that the pooled sensitivity of EPDS (Se = .80) for a subgroup of adult and non-HIV positive pregnant women was higher than that for HIV positive women (Se = .78). Nonetheless, one may not clearly ascertain from this review the extent to which HIV status of pregnant women influenced validity of screening instruments.

In this review, it was clear that in Mexico, sensitivity of EPDS among teenager pregnant women was 0.05 lower than its sensitivity among adult pregnant women [36, 37]. This may suggest that the population sampled may influence validity of a screening instrument. Studies have found that instruments may have different levels of sensitivity and specificity when applied to women at different stages of pregnancy. In this review, the EPDS had both highest sensitivity (Se = 1.0) [4] and lowest sensitivity (Se = .69) [34] among third trimester pregnant women and BDI had different sensitivity values among second trimester pregnant women in Brazil [1, 39]. It was however not possible in this review it establish whether screening instruments may have different levels of sensitivity and specificity when applied to women at different stages of pregnancy due to inconsistencies in completeness of reporting in original studies.

Lastly, while systematic reviews are widely recognised as an efficient, reliable and comprehensive source of evidence for decision-making, few systematic reviews have considered effects on health equity [14]. In the light of this, the reviewers’ recommendations were focused on the appropriate end-users (antenatal services in low resource settings) and we recognise that the findings are context-specific [14]. In this context, the EPDS emerged as the most suitable instrument for screening antenatal depression in low resource settings where time and other resources are limited. This performance of the EPDS in low resource settings is important as it supports the existing evidence from HICs which cannot always be applied effectively in low resource settings [53]. As such, this *emic* evidence will supplement the existing *etic* evidence to bring transformational health changes in antenatal care in low resource settings [13] which have heavy workloads, insufficient staff, poor funding and lack of medicines and supplies [11].

### Strengths and limitations

One of the key strengths of the review is the specific evidence on screening tools used in antenatal services in low resource settings. It may serve as an efficient, reliable and comprehensive source of evidence for decision-makers in low resource settings [14] since most evidence, generated from HICs, may not be applicable in low resource settings. A limitation of this review is that restrictions on language and date limits may have resulted in missing out some relevant articles.

## Conclusion

This review suggests that the EPDS can be a suitable instrument of preference for screening antenatal depression in low resource settings because its level of accuracy ranged from moderate to high in various settings. The EPDS is an easy and cheap tool for clinicians to administer during antenatal attendances and can help in identifying pregnant women at risk of depression [39].

## Abbreviations

AUC: Area under curve
BDI: Beck depression index
CES-D 20: Centre for epidemiologic studies depression scale 20
CI: Confidence interval
CIDI: Composite international diagnostic interview
CINAHL: Cumulative index to nursing and allied health literature
DSM-IV: Diagnostic and statistical manual of mental disorders version 4
EPDS: Edinburgh postnatal depression scale
HAM-D: Hamilton rating scale for depression
HICs: High income countries
HIV: Human immunodeficiency virus
HSCL-15: Hopkins symptoms checklist 15
HSCL-25: Hopkins symptoms checklist 25
ICD-10: International classification of diseases version 10
K-10: Kessler psychological distress scale
LMICs: Low and middle income countries
MINI: Mini-international neuropsychiatric interview
PICOS: Participants index test comparator test outcome measures study setting
QUADAS: Quality assessment of diagnostic accuracy studies
SCID: Structured clinical interview for DSM-IV
SRQ: Self-reporting questionnaire
STARD: Standards for the reporting of diagnostic accuracy studies
UMICs: Upper middle income countries
